# Identifying rare variant associations in population-based and family-based designs

**DOI:** 10.1186/1753-6561-8-S1-S58

**Published:** 2014-06-17

**Authors:** Asuman S Turkmen, Shili Lin

**Affiliations:** 1 Department of Statistics, The Ohio State University, 1179 University Drive, Newark, OH 43055, USA; 2 Department of Statistics, The Ohio State University, 1958 Neil Avenue, Columbus, OH 43210, USA

## Abstract

For almost all complex traits studied in humans, the identified genetic variants discovered to date have accounted for only a small portion of the estimated trait heritability. Consequently, several methods have been developed to identify rare single-nucleotide variants associated with complex traits for population-based designs. Because rare disease variants tend to be enriched in families containing multiple affected individuals, family-based designs can play an important role in the identification of rare causal variants. In this study, we utilize Genetic Analysis Workshop 18 simulated data to examine the performance of some existing rare variant identification methods for unrelated individuals, including our recent method (rPLS). The simulated data is used to investigate whether there is an advantage to using family data compared to case-control data. The results indicate that population-based methods suffer from power loss, especially when the sample size is small. The family-based method employed in this paper results in higher power but fails to control type I error. Our study also highlights the importance of the phenotype choice, which can affect the power of detecting causal genes substantially.

## Background

With rapid advances in genotyping technologies, it has becoming increasingly feasible to efficiently sequence large number of individuals, which allows us to assess the role of rare variants in influencing complex traits. However, because each rare variant is present in only a small number of individuals, standard association tests designed for common variants have low power to identify rare variants associated with the trait. This fact has led to the development of new statistical tests specifically targeting rare variants.

One may classify existing rare variant association methods into 2 categories: burden and nonburden tests. Burden tests, such as SUM [1] and combined multivariate and collapsing (CMC) [2], are based on the idea of collapsing/aggregating effects of rare variants within a region and they implicitly assume that all variation affecting phenotype acts in the same direction and that all (or a majority) of the variants within the region are causal. Given these limitations of burden-based methods, several other methods, such as sequence kernel association test (SKAT) [3], which build upon the kernel machine regression framework, have been proposed. However, nonburden tests lose power in the situations where burden tests are optimal. Consequently, a data-adaptive test called SKAT-O [4] that includes both burden tests (ie, SUM) and SKAT as special cases has been proposed.

Recently, we proposed a 2-step method rare variant Partial Least Squares (rPLS) [5] to reveal possible genetic effects related to both rare and common variants in population-based designs. This approach can be considered as a burden test that is robust to the presence of both deleterious and protective variants, as well as to the existence of noncausal variants within a genomic region being tested. We can apply the rPLS on any trait (quantitative or categorical) and at the same time have the flexibility of including non-single-nucleotide variant (SNV) covariates and of adjusting for population stratification as extra terms in the (generalized) linear model framework.

An important question that has not yet been addressed fully is the relative power of designs based on families and designs using unrelated individuals for identifying rare disease variants. All of the aforementioned methods that have been evaluated extensively are applicable to unrelated samples only. Utilizing the opportunity provided by Genetic Analysis Workshop 18 (GAW18), we compare the power of a family-based method, the modified family-based association test (FBAT)-v [6], for detecting rare variants' effects with the power of a number of population-based methods described above, including SKAT, SKAT-O, SUM, and rPLS. We further investigate the effect on power of various phenotypes defined on the same set of blood pressure measurements.

## Methods

### Data

The GAW18 data contain genotypes for odd-numbered chromosomes from a real human whole genome sequencing study with phenotypes at 4 time points and 200 replicates of the simulated longitudinal phenotype data. In this study, we consider only genotypes on chromosome 3. We analyzed 1389 individuals in 20 pedigrees, ranging from 27 to 107 individuals, for the family design. One hundred and forty-two unrelated individuals having observed genotypes were extracted from these pedigrees and used for population-based analysis. Systolic blood pressure (SBP) and diastolic blood pressure (DBP) measurements at the first time point were taken as quantitative traits. An individual with SBP ≥140 mm Hg or DBP ≥90 mm Hg, or who was on antihypertensive medications at that examination was classified as a case. The resulting binary trait corresponding to hypertension (HTN) disease status was included in the analysis. Because age and sex are known to influence blood pressure, we also included them as covariates in the analyses.

### Identification of rare variants for population and family data notation

Suppose that genotypes for *p *SNVs (common and rare) in a gene or a genomic region are available with *x_ij _*= 0, 1, or 2 for *i *= 1,2,...,n and *j *= 1,2,...,p coding for the number for minor alleles at locus *j *for individual *i*. The goal is to test whether the variants within candidate gene are associated with trait *y*. In this section, the approach proposed for population data (rPLS) and other existing methodologies employed in the paper is briefly described.

### Population-based tests

#### rPLS

The 2-step procedure rPLS [5] has been proposed for finding an optimum linear combination of the variants within a gene so that one can detect associations that are too weak to be detected for individual variants. The procedure starts with an initial variant selection step before the information is aggregated in the gene. In this study, we consider the following generalized linear regression model:

(1) $$g\left(\mu_{i}\right)=\beta_{0}+x_{i}\beta +z_{i}\gamma ,\;\;\;i=1,2,\ldots ,n$$

where μ_i _= E(y_i_), g is a known link function such as the identity link for continuous and logit link for binary trait; β_0 _is the intercept; *x_i _*= [x_i1_, x_i2_,..., x_ip_] is the vector of p variants from individual i and *β *is the corresponding p × 1 unknown coefficient vector; *z_i _*= [z_i1_, z_i2_,..., z_iq_] is the vector of q non-SNV covariates for individual i; and *γ *is the corresponding q × 1 unknown coefficient vector.

In the first step of rPLS, the elastic net (EN) estimator of the *β *is obtained to determine a subset of SNVs with nonzero coefficient estimates. The corresponding n × k matrix of genotypes is denoted by where *k *(≤p) is the number of SNVs selected by EN.

In the second step, we construct a supervariant, *t*, as an optimal linear combination of the variants in using partial least squares (PLS) [7]. Because *t *is a vector of size n × 1 and it summarizes genotype information, we can test the significance of β^* ^in the model

(2) $$g\left(\mu_{i}\right)=\beta_{0}+t_{i}\beta^{*}+z_{i}\gamma .\;i=1,2,\ldots ,n$$

The *p *value calculated for testing ${\text{H}}_{0}:\beta^{*}=0$ against ${\text{H}}_{\text{A}}\text{: }\beta^{*}\neq 0$ can be used to assess whether the trait and the gene (or genomic region) of interest are associated. Pros and cons of the method are briefly discussed in the Conclusion section. We refer the interested reader to Turkmen and Lin [5] for further details of the rPLS approach.

#### Other approaches for unrelated individuals

The sum test (SUM) [1] summarizes information across multiple SNVs with only 1 degree of freedom (DF) by creating a supervariant that is the sum of the number of minor alleles of all SNVs and tests association between the supervariant and the trait. As a kernel machine-based test, SKAT aggregates genetic information across the region using a kernel function and uses a variance component test for association. The fact that both classes of tests are optimal in certain conditions and the underlying biological mechanisms are often unknown creates a need for a data-adaptive test that is optimal for both scenarios. Consequently, a new class of tests called SKAT-O [4] was proposed. The test statistic for SKAT-O is an arbitrary linear combination of burden test (SUM) and nonburden test (SKAT), with SKAT-O identifying the optimal test within this class to maximize power.

### Family-based test

The FBAT-v method is among the first set of tests incorporating the collapsing model into pedigree-based analysis [6]. Two schemes are used: (a) FBAT-v0, which uses the collapsed sum of all the individual SNV contributions, and (b) FBAT-v1, which is the same as FBAT-v0 except that each SNV contribution is weighted inversely by its SNV frequency. Only rare variants (with minor allele frequency [MAF] less than 0.01) within the region analysed.

### Software

We carried out our analyses using R packages glmnet, SKAT, and plsgenomics, which were downloaded from http://cran.r-project.org/. FBAT analysis was done using *FBAT *software (FBAT v2.0.4 beta1). All tuning parameters needed in the implementation of the methods were set to their default values.

## Results

In this section we report association analyses of 1 binary trait (HTN), 2 quantitative traits (DBP, SBP), and the first principal component (PC1) obtained from these 2 quantitative traits with 1,002,216 SNVs on chromosome 3. The MAF distribution was severely skewed toward low-frequency alleles.

Using the base-pair locations of the SNVs, we assigned each SNV to a gene based on RefGene sequence records from the UCSC Golden Path. This procedure assigned 432,542 SNVs into a total of 1057 genes and the median number of SNVs within the genes was approximately 430. Among them, 1014 genes contain between 1 and 2000 SNVs, while the number of SNVs for the remaining 43 genes ranges from 2023 to 10,144. In the simulating model, 27 and 21 of 1057 genes are assumed to be associated with DBP and SBP, respectively. The union of these genes (yielding 30 genes given that there are 18 causal genes that are associated with both SBP and DBP) was considered to be associated with HTN and PC1. We carried out analyses using rPLS, SKAT, SKAT-O, SUM, and FBAT-v methods for variants in each gene using a gene-based approach.

### Unrelated individuals

The top causal variants for the simulated phenotypes DBP and SBP on chromosome 3 are in the gene *MAP4*. Consequently, we first explored the performance of population- based tests for detecting *MAP4*. Quantitative traits DBP, SBP, and PC1 were analyzed. Table 1 summarizes the results based on 200 replicates. Power is defined as the percentage of 200 replicates for which *MAP4 *is found to be significant, whereas the type I error is the average percentage of all noncausal genes that are found to be significant, that is, (total number of false positives)/[(number of noncausal genes)*200], where the number of noncausal genes is 1030, 1036, and 1027 for the traits DBP, SBP, and PC1, respectively. These calculations were done using the nominal α = 0.001 threshold. Table 1 indicates that SKAT and SKAT-O methods were either liberal (eg, SBP) or conservative (eg, DBP) depending on the considered trait, yet failed to detect *MAP4 *in any of the replicates. The SUM test provided higher power compared to SKAT and SKAT-O, with a slight increases in type I errors. On the other hand, rPLS had the highest power to detect *MAP4 *for all 3 quantitative traits, while type I errors were slightly elevated.

**Table 1:** Power and type I error for population-based methods at α = 0.1%

	DBP		SBP		PC1	
			
Method	Power	Type I	Power	Type I	Power	Type I
SKAT-O	0%	0.06%	0%	0.12%	0.50%	0.10%
SKAT	0%	0.05%	0%	0.12%	0.10%	0.10%
SUM	3%	0.12%	1.5%	0.12%	0%	0.12%
rPLS	13.5%	0.14%	20.5%	0.14%	14%	0.13%

Table 2 provides a more global view for the performance of the population-based tests by examining frequency of successful identifications for each causal gene. Because SKAT and SKAT-O performed at par, we have only included results for SKAT-O. Here, a gene is listed if it was detected at least 10 times out of 200 by any method for the traits SBP, DBP, or PC1. The genes listed in Table 2, except *SCAP*, are causal for all traits, which is associated only with SBP. *SEMA3F *is a notable gene that could not be detected by any of the methods for the trait DBP but was detected 68 times with rPLS when PC1 was used. Four causal SNVs within *SEMA3F *have moderate and similar effect sizes for DBP and SBP. Therefore, combining information in DBP and SBP using the first PC seems to strengthen the signal and lead to higher power for detecting the association. Overall, Table 2 also demonstrates that the rPLS method is the most powerful compared to the other population-based methods, while having a slightly larger type I error that is still quite close to the nominal level.

**Table 2:** Number of times that causal genes were detected (at least 10 times) out of 200 replicates by any method

	DBP			SBP			PC1		
			
Gene	SKAT-0	SUM	rPLS	SKAT-0	SUM	rPLS	SKAT-0	SUM	rPLS
*MAP4*	0	6	27	0	3	41	1	0	28
*SEMA3F*	0	0	0	0	0	3	0	6	68
*SCAP*	0	0	0	2	0	16	0	0	0

Another important feature of the rPLS is its ability to identify the most important SNVs within a genomic region, a unique feature not available in the other methods. This can be done by simply ranking the weights (components of the first PLS loading vector) employed to find optimum linear combination. For instance, when we considered the SBP trait for replicate 5 in which *MAP4 *was detected, the ranking of the loadings indicated that causal SNV *3_48040284 *had the largest value.

### Family-based versus population-based design

For the family-based study, only 2 traits (HTN with 30 causal genes and SBP with 21 causal genes) and the fifth replicate were considered because of the computational burden. In addition to the causal genes, 50 noncausal genes were randomly selected to gauge type I error and accuracy rates that were calculated at α = 0.1%. Here, the type I error is equal to the number of significant unassociated genes divided by 50, while the power to detect association with SBP (HTN) is equal to the number of significantly associated genes divided by 21 (30). Accuracy is defined as the proportion of true results (both true positives and true negatives).

As Table 3 indicates, none of the population-based methods was able to detect association with HTN, but they had zero type I error. rPLS is the only population-based method that identifies one of the causal genes (ie, *MAP4*) for SBP, so the highest accuracy was achieved by rPLS. Although 2 versions of FBAT-v give larger powers, they had inflated type I errors.

**Table 3:** The power, type I error, and accuracy results for the fifth replicate traits HTN and SBP at α = 0.1%

	HTN (30C, 50NC)			SBP (21C, 50NC)		
			
Method	Type 1	Power	Accuracy	Type 1	Power	Accuracy
FBAT-v0	4.00%	3.33%	61.25%	10.00%	9.52%	66.20%
FBAT-v1	2.00%	3.33%	62.50%	6.00%	9.52%	69.01%
SKAT-0	0.00%	0.00%	62.50%	0.00%	0.00%	70.42%
SKAT	0.00%	0.00%	62.50%	0.00%	0.00%	70.42%
SUM	0.00%	0.00%	62.50%	0.00%	0.00%	70.42%
rPLS	0.00%	0.00%	62.50%	0.00%	4.76%	71.83%

C, causal genes; NC, noncausal genes.

## Conclusions

Our analyses based on unrelated individuals have found *MAP4, SEMA3F*, and *SCAP *genes to be associated with the traits for at least 10 replicates when rPLS is employed. It has been shown that the choice of the trait can affect the power of the test regardless of the methodology used. rPLS was the most powerful among the population-based tests without considerably elevated type I error. In general, the initial variant screening step in rPLS can lead to an elevated type I error, but as shown by Turkmen and Lin [5], the increase in type I error becomes negligible when the signal-to-noise ratio decreases (ie, more noncausal variants are present in the gene than causal ones), which is a realistic scenario encountered in applications. SKAT and SKAT-O were much less consistent in terms of controlling type I error, while the SUM test was always liberal. Overall, rPLS has the advantage of a lesser DF than the burden tests, while it does not depend on the strict assumption that all SNVs have common effect size and direction, nor does it need to use a rare variant threshold. Furthermore, the optimal projection vector obtained in rPLS can be used to determine which SNVs within a gene are more important by quick evaluation of magnitudes, a feature not available in other methods.

FBAT-v yielded higher powers compared to results based on unrelated individuals but failed to control type I error rate. This is most likely a result of the existence of a large number of noncausal rare variants within the region. As mentioned in the original paper [6], linkage disequilibrium between causal and noncausal SNVs and lack of normality by restricting to rare variants could be other factors that are responsible for the inflated false positives.

In conclusion, although family-based methods can be more powerful because they can make fuller use of available data, further research is needed to find ways to control the type I error rate. Absent a more appropriate family-based method, rPLS emerges as a viable alternative for analyzing population-based data.

## Competing interests

The authors declare that they have no competing interests.

## Authors' contributions

AST and SL conceived the project, designed the algorithm and wrote the manuscript. AST implemented the algorithm and analyzed the data. SL supervised the research and polished the manuscript. Both authors read and approved the final manuscript.

## References

[B1] Pan W Asymptotic tests of association with multiple SNPs in linkage disequilibrium Genet Epidemiol 2009 33 497 507 10.1002/gepi.20402 19170135 PMC2732754

[B2] Li B Leal SM Methods for detecting associations with rare variants for common diseases: application to analysis of sequence data Am J Hum Genet 2008 83 311 321 10.1016/j.ajhg.2008.06.024 18691683 PMC2842185

[B3] MC Wu S Lee T Cai Y Li Boehnke M Lin X Rare variant association testing for sequencing data using the sequence kernel association test (SKAT) Am J Hum Genet 2011 89 82 93 10.1016/j.ajhg.2011.05.029 21737059 PMC3135811

[B4] Lee S Wu MC Lin X Optimal tests for rare variant effects in sequencing association studies Biostatistics 2012 13 762 775 10.1093/biostatistics/kxs014 22699862 PMC3440237

[B5] Turkmen AS Lin S An optimum projection and noise reduction approach for detecting rare and common variants associated with complex diseases Hum Hered 2012 74 51 60 10.1159/000343797 23154579

[B6] Yip WK De G Raby BA Laird N Identifying causal rare variants of disease through family-based analysis of Genetic Analysis Workshop 17 data set BMC Proc 2011 5 suppl 9 S21 10.1186/1753-6561-5-S9-S21 22373204 PMC3287856

[B7] De Jong S SIMPLS: an alternative approach to partial least squares regression Chemometr Intell Lab Syst 1993 18 251 263 10.1016/0169-7439(93)85002-X

